# When fandom becomes a problem: Development of the Problematic Celebrity Fanship Scale using a representative adult sample

**DOI:** 10.1186/s40359-026-04064-w

**Published:** 2026-02-04

**Authors:** Ágnes Zsila, Lynn E. McCutcheon, Reza Shabahang, Mara S. Aruguete, Róbert Urbán, Ágnes Buvár, Rita Horváth, Zsolt Demetrovics

**Affiliations:** 1 Institute of Psychology, ELTE Eötvös Loránd University, Budapest, Hungary; 2 North American Journal of Psychology, Winter Garden, FL USA; 3 Flinders University Institute for Mental Health and Wellbeing, College of Education, Psychology and Social Work, Flinders University, Bedford Park, Adelaide, SA Australia; 4 Lincoln University, Missouri, MO USA; 5 Institute of People-Environment Transaction, ELTE Eötvös Loránd University, Budapest, Hungary; 6 Doctoral School of Psychology, ELTE Eötvös Loránd University, Budapest, Hungary; 7 Centre of Excellence in Responsible Gaming, University of Gibraltar, Gibraltar, Gibraltar

**Keywords:** Behavioral addiction, Celebrity worship, Problematic celebrity fanship, Scale development

## Abstract

**Background:**

There has been considerable research interest in the personality characteristics and mental health of individuals showing excessive levels of celebrity admiration. However, the conceptualization and measurement of potentially problematic levels of engagement with celebrities remain unclear. This study introduces and operationalizes the concept of problematic celebrity fanship within the theoretical framework of behavioral addictions. The main aim is to develop a measurement instrument to assess problematic celebrity fanship.

**Methods:**

Participants were 755 individuals with a favorite celebrity (51.4% men, *M*_age_ = 36.4 years, *SD* = 13.4), derived from a representative sample of Hungarian adults (*N* = 2,028).

**Results:**

The 8-item unidimensional Problematic Celebrity Fanship Scale (PCFS) demonstrated sound psychometric properties across factor structure, measurement invariance, reliability, and validity. Based on latent profile analysis, a cutoff score of 26 was suggested. Prevalence rate for problematic celebrity fanship was 1.4% (adults aged 18–64 years); however, young adults (aged 18–34 years) showed a higher, 2.3% prevalence. Problematic celebrity fanship severity was moderately associated with psychological distress, lower self-concept clarity, problematic Internet use, and celebrity worship.

**Conclusions:**

The PCFS can assist in the recognition and assessment of problematic celebrity fanship in research and practice by identifying individuals experiencing mental health difficulties in relation to their excessive engagement with a celebrity.

**Supplementary Information:**

The online version contains supplementary material available at 10.1186/s40359-026-04064-w.

## Background

The tendency to follow admired celebrities has generated much research attention in the past two decades [1]. Although there is a lack of consensus on how to conceptualize and operationalize the socio-emotional relationship between celebrities and fans, studies suggest that some individuals are more prone to becoming attracted to an admired celebrity than others [2, 3]. Drawing on the phenomena of parasocial relationships (i.e., one-sided socio-emotional intimacies between viewers and media figures, usually viewed as positive bonds; [4, 5]) and fan motivations [6], celebrity worship has been conceptualized as an excessive parasocial engagement with an admired celebrity, comprising healthy behaviors (e.g., seeking the company of individuals sharing the same enthusiasm for a celebrity), which can sometimes progress into problematic levels of engagement (e.g., feeling compelled to learn the personal habits of a celebrity [2, 3]). Problematic or excessive levels of celebrity worship are often characterized by impulsive and compulsive features on affective, cognitive, and behavioral levels, which have been associated with poor mental health and weak social relationships [1]. The present study seeks to re-conceptualize and operationalize problematic levels of celebrity fanship within the current theoretical framework of behavioral addictions [7, 8].

Existing measures of celebrity attitudes lack a clear distinction between mild and extreme forms of celebrity attraction. For example, the Idol Worship Questionnaire [9] and the Expression of Idolization Scale [10] assess both general and extreme expressions of fanship without a clear distinction between healthy and unhealthy engagement with a celebrity. Moreover, the terms “worship” and “idolization” suggest excessive engagement even for mild levels of interest in a celebrity. Therefore, the present authors use a more neutral term, “celebrity fanship,” to describe broader levels of fan engagement. The most commonly used assessment instrument [1], the Celebrity Attitude Scale (23-item CAS; [2, 3]) distinguishes between healthy (i.e., entertainment–social) and excessive (i.e., intense–personal and borderline–pathological) levels of celebrity worship, but defines the comprehensive construct (including the increasingly intense levels) simply as celebrity worship, leading to a slight conceptual confusion [11]. Accordingly, the short version of the CAS (CAS–7; [12]) has an established cutoff threshold to identify “celebrity worshipers” based on the total score with healthy and problematic dimensions merged. Therefore, there remains a need for a measure specifically assessing the problematic aspects of celebrity fanship.

Regarding the terminology of the present study, celebrity fanship can be viewed as an enthusiasm and dedication towards a celebrity, characterized by typical fan activities (e.g., following the news about the celebrity, creating fan art), similar to the entertainment–social dimension of celebrity worship. Problematic celebrity fanship can be defined as an excessive form of engagement with a celebrity, which can lead to harm in the individual’s professional and personal life. Therefore, a key difference between intense–personal, borderline–pathological celebrity worship and problematic celebrity fanship is that excessive forms of celebrity worship involve deep emotional engagement, strong identification with the celebrity, and extreme behaviors (e.g., spending large amounts of money on celebrities’ personal possessions), whereas problematic celebrity fanship can be described by symptoms typically associated with behavioral addictions (e.g., unsuccessful attempts to spend less time dedicated for fan activities).

The Absorption–Addiction Model [2, 3] describes the development of excessive celebrity worship, proposing that individuals with weaker identity structures (e.g., self-concept clarity) are more prone to become absorbed in celebrities in order to strengthen their identities through identification with an admired celebrity. This identification process can become excessive in some cases, leading to the manifestation of addiction-like symptoms (e.g., preoccupation, loss of control). Early studies (e.g., [13, 14]) investigated celebrity worship tendencies within a personality framework, demonstrating that entertainment–social celebrity worship is more closely associated with extraversion, while the intense–personal dimension is rather associated with neuroticism, and the borderline–pathological level with psychoticism. Maltby et al. [14] also hypothesized a positive association of weaker ego-identity (i.e., doubts in own decisions and skills, and inability for responsibility, [15]) and higher tendency for obsessive-compulsive disorder (i.e., a condition characterized by obsessive, undesired, and uncontrollable thoughts [e.g., fear of contamination, sexual thoughts] and behaviors [e.g., checking, washing]; [16]) among those scoring higher on the intense–personal dimension of celebrity worship. However, Maltby et al. [14] found that more symptoms of obsessive-compulsive disorder and weaker ego-identity were only weakly associated with the two problematic dimensions of celebrity worship (i.e., intense–personal and borderline–pathological), suggesting that celebrity worship may be more appropriately contextualized within other psychological disorders.

Several studies (see [1, 17]) highlighted similarities in the features of celebrity worship and behavioral addictions, such as impulsive behaviors (e.g., willingness to do something illegal if the celebrity requested it). Therefore, additional research on the link between excessive levels of celebrity fanship and behavioral addictions is warranted. Behavioral addiction is an umbrella term describing a problematic behavior with compulsive and impulsive features, which leads to negative consequences in various life domains, such as work or academic commitment, leisure activities, and social relationships [7, 8, 18]. Empirical evidence demonstrates positive associations between celebrity worship and behavioral addictions (e.g., problematic Internet and social media use, compulsive buying, gambling addiction; see [19] for a review), in addition to psychoactive substance use-related excesses [20]. Moreover, celebrity worship has been associated with psychological distress (see [17] for a review) and weak interpersonal relationships [1], similar to the negative consequences experienced in relation to behavioral addictions [21].

Recently, He and Liu [22] investigated celebrity worship in a behavioral addiction theoretical framework, identifying overlapping features between the constructs, such as functional impairment (e.g., jeopardizing social relationships, work or school commitments due to excessive engagement with a celebrity), mood modification (e.g., feeling comfortable when immersing oneself in the updates of a favorite celebrity), or withdrawal (e.g., feeling anxious and restless when unable to access the celebrity online), and developed an assessment instrument to operationalize excessive celebrity worship. Although the Excessive Celebrity Worship Behavior Questionnaire demonstrated good psychometric properties, its complexity and length are not typical in the measurement of behavioral addictions (see [23–25]). Furthermore, scale development was based on netnography and interviews with fans, lacking a clear theoretical model. Additionally, some factors extended beyond the scope of both celebrity worship and behavioral addictions (e.g., replacement of real with virtual social relationships, eating and sleep disorders). Although such extensions can enrich prior theoretical foundations with timely aspects, more evidence may be needed to determine whether these aspects are integral parts of the phenomenon, or comorbid disorders (e.g., excessive buying). Moreover, eating and sleep disorders are often treated as consequences of behavioral addictions instead of being components of a specific addiction (see [26, 27]).

The present study aimed to address the limitations in the measurement of problematic celebrity fanship by developing a brief but comprehensive, theoretically grounded, and psychometrically sound assessment instrument. Celebrity worship, parasocial relationship, technology-related behavioral addictions (i.e., problematic Internet use and problematic social media use), and psychological distress were also assessed to support the validity of the measurement instrument. The scale was designed to enable the identification of individuals at risk of problematic celebrity fanship. Prevalence rates were estimated in a representative sample of adults to contribute to a broad generalizability of findings.

## Methods

### Participants and procedure

The present sample of fans was derived from a sample of Hungarian adults aged 18 to 64 years, which was representative of the general adult population of Hungary according to gender, age, location, and size of residence (*N* = 2,028; 50.5% men, 49.5% women, *M*_age_ = 37.9, *SD* = 13.3). The target sample size was 2,000 participants; however, the young adult population (aged 18–34) years was oversampled to reach the individual sample size of 1,000. Prevalence rates were calculated for this subsample besides the total sample, as young adults could be more affected by celebrity fanship [12]. Therefore, the theoretical margin of error in the total sample of adults aged 18–64 years was estimated at ± 2.2%, while it was ± 3.1% among young adults aged 18–34 years, resulting in a total sample of 2,028 participants. Exclusion criteria were [1] not having a favorite celebrity (*n* = 1,156) [2], not providing a valid answer to the name of the favorite celebrity (*n* = 90), and missing data in at least half of the newly developed scale items (*n* = 27). Therefore, the final sample consisted of 755 participants (51.4% men, 48.6% women, *M*_age_ = 36.4 years, *SD* = 13.4, range: 18–63 years). More than half of the participants lived in a town (*n* = 403; 53.38%), while 18.81% (*n* = 142) resided in the capital city, and 27.81% (*n* = 210) lived in a village. Regarding education, the majority of participants completed secondary school education (*n* = 521; 69.01%), while 16.42% (*n* = 124) completed eight primary school classes or less, and 14.57% (*n* = 110) had a BA or MA diploma. Favorite celebrities were mostly musicians (44.1%), actors (22.8%), and athletes (19.9%), showing a large variety of preferences (i.e., none of the indicated celebrities were named as favorites by at least 5% of the sample). A computer-assisted personal interviewing (CAPI) method with computer-assisted self-interviewing (CASI) elements was employed by professional interviewers. In more detail, interviewers verbally asked each question or scale item and recorded the respondents’ answers on a computer. However, the computer was turned to the respondent for the administration of sensitive questions, which were thereby self-administered. This method enables higher data accuracy and increased validity, as participants can seek assistance in real time, directly from professional interviewers if questions arise. Mixed (i.e., CAPI with CASI) methods also enable faster data processing compared to traditional paper-and-pencil administration or video/voice recordings during personal interviews. Participants provided informed consent. Ethical approval was obtained from the Institutional Review Board of the lead author’s former university, before the data collection (protocol number: 2023_69).

## Measures

### Scale development protocol for the problematic celebrity fanship scale (PCFS)

The purpose of the present scale development was to provide a brief and concise assessment instrument to measure problematic celebrity fanship within a behavioral addiction theoretical framework (see SM 1). Initially, the expert panel of the research team, consisting of researchers with significant experience in behavioral addictions (*n* = 3) and celebrity worship (*n* = 3), reviewed current theoretical frameworks and diagnostic criteria for behavioral addictions, such as gambling disorder (based on the suggested diagnostic criteria listed in the fifth edition of the Diagnostic and Statistical Manual of Mental Disorders [DSM-5; [28, 29]]), gaming disorder (based on the suggested criteria included in the 11th edition of the International Classification of Diseases [ICD-11, [30]]), and formerly, suggested criteria in the DSM-5 Section III for Internet gaming disorder), and problematic Internet use, problematic social media use, and problematic pornography use (based on the commonly applied 6-components model of addictions within the biopsychosocial framework by Griffiths [31]).

First, the research team identified the diagnostic criteria and components of behavioral addictions. Second, symptoms and components that could be applied to celebrity fanship were selected (see a structured overview of the criteria in Table 1).

**Table 1:** Overview of the symptoms of behavioral addictions considered as a basis for the construction of the components of the Problematic Celebrity Fanship Scale (PCFS)

Component of problematic celebrity fanship	Gaming disorder (ICD-11); [30] and Internet gaming disorder (DSM-5; [28, 29])	Gambling disorder (DSM-5; [28, 29])	Components model (Griffiths [31])
Preoccupation	Preoccupation with gaming	Preoccupation with gambling (e.g., having recurrent thoughts of past gambling activities)	Preoccupation; the activity becomes the most important aspect of the persons’ lives, dominating their thoughts (salience)
Withdrawal	Experiencing undesired symptoms when gaming is ceased (withdrawal)	Becoming restless or irritable when attempting to stop or reducing gambling activity	Experiencing undesired physical or psychological effects when the activity is reduced or ceased (withdrawal symptoms)
Tolerance	Needing to spend more and more time gaming (tolerance)	Needing to gamble with more money to gain the desired level of satisfaction	Needing to increase participation in the activity to achieve previous levels of effect (tolerance)
Unsuccessful attempts	Unsuccessful attempt to control the gaming activity (loss of control)	Unsuccessful attempt to control or stop the activity	
–			Returning to previous patterns of use after short or longer periods of control/abstinence (relapse)
–		After losing significant amounts of money due to gambling, returning to “chase” losses	
–		Relying on others financially to compensate for the losses due to gambling	
Continuation	Continued excessive use despite knowledge of psychosocial problems (continuation)		
–	Deception of others regarding the gaming activity (deception)	Lying to others to conceal the extent of engagement with gambling activity	
Escape and mood modification	Using games to escape from negative mood and relieve negative feelings (escape)	Gambling when feeling distressed (e.g., depressed, anxious)	Subjective experiences of feeling “high”, “escape”, and being in an elevated mood (mood modification)
Negative consequences and conflicts	Jeopardizing or losing relationships, work/academic opportunities (negative consequences)	Jeopardizing or losing relationships, work/academic opportunities	Conflict between the persons and their environments due to the activity, such as deterioration of personal relationships, work/academic achievements, or other recreational activities (conflict)
Loss of interest in other activities	Loss of interest in other activities		

*ICD* International Classification of Diseases, *DSM* Diagnostic and Statistical Manual of Mental Disorders

All of the proposed diagnostic criteria of gaming disorder could be applied to the concept of celebrity fanship, except for deception. Regarding the 6-component model by Griffiths [31], the component of relapse includes shorter or longer periods of abstinence or reduced activity. As similar patterns (i.e., reducing the engagement with a favorite celebrity on purpose, and finally returning to previous levels of engagement) have not been reported with regard to celebrity admiration in the literature, this component was consensually excluded from further consideration. Unsuccessful attempts to reduce the activity without this specific relapse pattern, originally observed in relation to smoking [31], were found to be more relevant to celebrity fanship by the expert panel. Likewise, financial reliance on others to support the problematic activity, and chasing financial losses as a compensation mechanism were found to be specific to gambling disorder, which could not be considered for celebrity fanship in which the role of financial support from others is still lacking empirical evidence. Finally, deceiving others (e.g., therapist, family members, or friends) to conceal the time and effort invested in the problematic activity was found to be less relevant in the present context, as the explicit expression of fan identity/activity (e.g., creating fan art, wearing band t-shirts) is common among fans, and over-identification with an admired celebrity was a core component of excessive celebrity worship in previous research (see the Absorption-Addiction Model by McCutcheon et al. [2, 3]). Therefore, motivation to lie about fan engagement may not be interpretable in this context. Overall, eight components were considered as a basis for item creation (see Table 1).

Subsequently, two items were created per component (*N* = 16 in total, see SM 2) by three expert members of the research team with significant experience in scale development. Subsequently, these items were carefully revised by the expert panel. The original Hungarian items were translated to English and back-translated to Hungarian by two independent translators in the research team with significant experience in cross-cultural validation of self-report scales. A commonly used rating scale (1 = *never*, 2 = *rarely*, 3 = *sometimes*, 4 = *often*, 5 = *almost always/always*) and time interval (past 12 months) were employed, derived from self-report scales assessing behavioral addictions such as the Problematic Online Gaming Questionnaire (POGQ; [32]), the Problematic Internet Use Questionnaire (PIUQ; [25]), and the Bergen Social Media Addiction Scale (BSMAS; [23]).

After evaluating the psychometric properties of the 16-item version of the Problematic Celebrity Fanship Scale (PCFS; see SM 2), a short, 8-item version was created based on statistical (i.e., higher factor loadings and lower inter-item correlations) and theoretical considerations (i.e., relevance and redundancy of the item content). Specifically, 6 items were selected based on both the content and higher factor loadings compared to the other item comprising the component, while 2 items were selected based on lower inter-item correlations or theoretical considerations. Specifically, the item “I became restless or irritable when I couldn’t watch/hear my favorite celebrity for as long as I wanted” (escape and mood modification) showed a particularly high inter-item correlation (*r* = 0.76) with the item “I have increased my time spent following the details of my favorite celebrity’s life” (tolerance), indicating redundancy. Moreover, the item “I fantasized more and more about my favorite celebrity” (tolerance) was found to be more general in content and showed extremely high correlation (*r* = 0.81) with the item “When I thought about my favorite celebrity, I forgot about everything else” (preoccupation). Therefore, the other item comprising the respective theoretical components was retained in these cases (see SM 2). The final items of the PCFS are presented in Table 2 and in SM 1 for use. Before administering the PCFS, participants were introduced to the general definition of a favorite celebrity based on McCutcheon et al. [3], describing a celebrity as a famous living person (or one who died during the participants’ lifetime) that the respondent greatly admires.

**Table 2:** Factor loadings and content of items comprising the Problematic Celebrity Fanship Scale (PCFS)

Item content	Component	EFA (*n* = 377)	CFA (*n* = 378)
1. My interest in my favorite celebrity has helped me forget about my real-life problems.	escape and mood modification	0.668	0.541
2. I got upset when something prevented me from seeing/hearing my favorite celebrity.	withdrawal	0.843	0.810
3. I have been unsuccessful at spending less time following my favorite celebrity’s life.	unsuccessful attempts	0.825	0.796
4. I have increased my time spent following the details of my favorite celebrity’s life (e.g., posts on social media).	tolerance	0.828	0.843
5. I had trouble getting interested in anything else besides my favorite celebrity.	loss of interest in other activities	0.844	0.877
6. My interest in my favorite celebrity has led to conflicts with others (e.g., parents, classmates).	negative consequences and conflicts	0.822	0.833
7. Despite being aware that my celebrity interest was negatively influencing my work/studies, I couldn’t stop it.	continuation	0.814	0.843
8. When I thought about my favorite celebrity, I forgot about everything else.	preoccupation	0.812	0.801

The instruction for the administration of the PCFS is as follows: “Please think back to the past 12 months and indicate on the following scale how often you experienced these things in relation to your favorite celebrity. 1 – never, 2 – rarely, 3 – sometimes, 4 – often, 5 – almost always/always”. To get a total score, individual item scores should be added. Total scores can range between 8 and 40. *EFA* exploratory factor analysis, *CFA* confirmatory factor analysis

### Other assessment instruments

Celebrity worship was assessed using the 7-item short version of the Celebrity Attitude Scale (CAS-7; [2, 12]), which contains two subscales: entertainment–social (3 items; α = 0.81) and intense–pathological (4 items; α = 0.88). Items are rated using a 5-point Likert-scale (1 = *strongly disagree*, 5 = *strongly agree*). Higher scores indicate greater celebrity admiration for entertainment–social and intense–pathological purposes. Moreover, a total score can be calculated by summing all items, which quantifies an overall celebrity admiration level (α = 0.88).

Parasocial relationships between fans and celebrities were assessed using the 8-item unidimensional Parasocial Interaction Scale (PSI-Scale; [33]). Items are rated using a 5-point Likert-scale (1 = *strongly disagree*, 5 = *strongly agree*). Higher scores indicate stronger parasocial relationships with favorite celebrities (α = 0.86). Items were translated from English to Hungarian and back-translated by two independent translators of the research team.

Psychological distress was assessed by the 9-item, 3-factor Depression, Anxiety, and Stress Scale (DASS-9; [34, 35]). Participants rated each item on a 4-point scale (0 = *did not apply to me at all*, 3 = *applied to me very much*,* or most of the time*). Due to the high inter-factor correlations in the present study (*r*s ranged from 0.72 to 0.75), items representing the symptoms of depression, anxiety, and stress were defined as a unidimensional construct reflecting psychological distress (α = 0.91), as recommended by Yusoff [35]. Higher scores indicate higher levels of psychological distress. Items were translated and back-translated by two independent translators of the research team.

Problematic Internet use was measured by the 9-item unidimensional Problematic Internet Use Questionnaire (PIUQ-9; [25, 36]). Participants rated the items on a 5-point scale (1 = *never*, 5 = *almost always*). Higher scores indicate higher problematic Internet use symptom severity (α = 0.93).

Problematic social media use was assessed by the 6-item unidimensional Bergen Social Media Addiction Scale (BSMAS; [23]). Items are rated on a 5-point scale (1 = *never*, 5 = *almost always*). Higher scores indicate higher problematic social media use symptom severity (α = 0.92).

Self-concept clarity was measured by the 12-item unidimensional Self-Concept Clarity Scale (SSC Scale; [37, 38]). Items are rated on a 5-point Likert-scale (1 = *strongly disagree*, 5 = *strongly agree*). Higher scores indicate higher self-concept clarity (α = 0.88).

### Statistical analysis

Data analysis was performed using SPSS 21.0 and Mplus 7.4 software [39]. Based on the Kaiser-Meier-Olkin (KMO) test result (KMO = 0.947), which reached the level of excellent sample adequacy (> 0.900; [40]) and the significant Bartlett’s [41] test result (*χ2* = 4,059.864, df = 28, *p* < 0.001), the present data was suitable for factor analysis.

First, factor structure of the PCFS was investigated by exploratory factor analysis (EFA) on the first half of the sample (*n* = 377), using geomin rotation after randomly half-splitting the total sample. Second, confirmatory factor analysis (CFA) was performed on the second half of the sample (*n* = 378), using a robust maximum likelihood estimator (MLR). Although applying a weighted least squares mean and variance adjusted (WLSMV) estimator is recommended for categorical or ordinal data [42], Rhemtulla et al. [43] found no substantial advantages of the use of a WLSMV estimator over the maximum likelihood (ML) method when variables included five or more categories and the sample size was relatively small. Therefore, there is no consensus on the common application of a WLSMV or ML/MLR estimator, as careful consideration is required in each case, based on the sample size, the nature of the data, and the specifics of the measures [43]. To increase transparency and replicability, the present CFA analyses were also conducted employing a WLSMV estimator (see SM 2 for the EFA and CFA for the 16-item version PCFS, SM 3 for the EFA and CFA for the 8-item version PCFS, SM 4 for the unstandardized factor loadings of the 8-item version of the PCFS, SM 6 for the CFA with covariates model, and SM 7 for the measurement invariance testing with a WLSMV estimator). No substantial differences were observed across the analyses with the two different estimators. Goodness of model fit was evaluated based on the commonly applied thresholds for sample size independent indices [42, 44]: Comparative Fit Index (CFI ≥ 0.95 for excellent, ≥ 0.90 for acceptable), Tucker-Lewis index (TLI ≥ 0.95 for excellent, ≥ 0.90 for acceptable), Root-Mean-Square Error of Approximation (RMSEA ≤ 0.06 for excellent, ≤ 0.08 for acceptable) with its 90% confidence interval, and Standardized Root-Mean-Square Residuals (SRMR ≤ 0.05 for excellent, ≤ 0.10 for acceptable). Although the chi-square (*χ2*) test and the degrees of freedom (*df*) are also commonly reported, the *χ2-*test result is sensitive to sample size and model misspecification issues [42, 45]; therefore, following the recommendation of Brown [42] and the practice by Tóth-Király et al. [46], only sample size independent model fit indices were evaluated in the present study. Reliability (Cronbach’s alpha and McDonald’s omega) was acceptable above 0.60 [47, 48]. Unstandardized factor loadings and residual variances are presented in SM 4.

Convergent and divergent validity of the PCFS was examined using a CFA with covariates model and its underlying correlation matrix of latent variables in which the associations of theoretically similar constructs representing the relationship with celebrities (i.e., celebrity worship and parasocial relationship), problematic behaviors (i.e., problematic Internet and social media use), and personality (i.e., self-concept clarity) and mental health indicators (i.e., psychological distress) with problematic celebrity fanship were investigated.

Gender invariance was tested using multigroup CFAs [49] to confirm that investigating gender differences in PCFS scores is meaningful. Therefore, gender (*1 = men*,* 2 = women*) was used as a grouping variable in the models with increasingly constrained parameters to detect potential measurement biases (i.e., configural, metric, scalar). Nonsignificant changes in the fit indices indicate measurement invariance in the examined levels [50–52]: ΔCFI ≤ 0.010; ΔTLI ≤ 0.010; and ΔRMSEA ≤ 0.015; ΔSRMR ≤ 0.030 for metric and 0.015 for scalar levels.

Latent profile analysis (LPA) was conducted on the final, 8-item PCFS to identify individuals potentially at risk of problematic celebrity fanship. The final number of classes based on the tested models was selected using the following fit indices: lower scores on the Akaike information criterion (AIC), bias-corrected Akaike information criterion (CAIC), Bayesian information criterion (BIC), and sample-size adjusted Bayesian information criterion (SSABIC); entropy value close to 1, and the Lo-Mendell-Rubin Adjusted Likelihood Ratio Test (LMR Test) for which a non-significant *p*-value (*p* > 0.05) indicates the appropriateness of the previous model with fewer classes [39]. Based on the LPA, sensitivity, specificity, positive (PPV) and negative predictive values (NPV) with overall accuracy were calculated to explore potential cut-off thresholds [53, 54].

Prevalence rates were estimated on weighted samples. To address slight biases and the oversampling of young adults (aged 18–34 years), a non-item-count matrix weighting by stratum (gender, age, location, and size of residence) was employed for the total sample (weighted *N* = 2,000). Furthermore, prevalence rates are reported separately for the young adult group (weighted *N* = 1,001) who may be more affected by the phenomenon under investigation. To avoid potential biases accounted for the weighting procedure, all analyses beyond prevalence estimations were performed on the unweighted dataset.

## Results

### Factor structure of the Problematic Celebrity Fanship Scale (PCFS)

First, EFA was conducted on the initial item pool consisting of 16 items (see SM 2) on the first subsample (*n* = 377). Only the unidimensional structure showed an eigenvalue above 1 (10.197, respectively), which yielded acceptable fit indices (see Table 3 ). Subsequently, CFA on the second subsample (*n* = 378) confirmed the unidimensional structure of the PCFS, with acceptable model fit. Modification indices were generally low, indicating no specific need for model improvement and suggesting no hidden factors. Final model fit and further psychometric characteristics are presented in Table 3 .

The internal consistency of the 16-item version PCFS was particularly high (α = 0.96), indicating that some items may be highly redundant or homogenous in content. Therefore, when creating a brief version of the PCFS, particular attention was paid to excluding redundant or general items, and items showing extremely high inter-item correlations. Finally, 8 items were selected for the brief version of the PCFS based on the previously described scale development protocol.

To ensure that the brief version of the scale is appropriate for broader use, the described psychometric analysis was performed on these 8 items, of which each represents a component of the original scale. EFA was conducted on the first subsample (*n* = 377). Again, only the unidimensional structure yielded an eigenvalue above 1 (5.568, respectively), which showed excellent fit (see Table 3). The subsequent CFA on the second subsample (*n* = 378) confirmed the unidimensional factor structure with excellent fit indices. Reliability indices and descriptive statistics are presented in Table 3. Further item-level descriptive statistics are reported in SM 5.

**Table 3:** Model fit indices, reliability, and descriptive statistics of the 8- and 16-item version of Problematic Celebrity Fanship Scale (PCFS)

	16-item version		8-item version	
	EFA	CFA	EFA	CFA
Model fit indices				
χ^2^ *(df)*	288.489 (104)***	332.481 (104)***	35.710 (20)*	31.569 (20)†
CFI	0.928	0.915	0.985	0.989
TLI	0.917	0.902	0.979	0.984
RMSEA (90% CI)	0.069 (0.059–0.078)	0.076 (0.067–0.086)	0.046 (0.019–0.070)	0.039 (0.004–0.064)
SRMR	0.042	0.050	0.025	0.022
Factor loadings (range)	0.648–0.845	0.568–0.866	0.668–0.844	0.541–0.877
Reliability				
Cronbach’s alpha	0.96		0.93	
McDonald’s omega	0.96		0.93	
Inter-item correlations (range)	0.41–0.81		0.45–0.74	
Item-total correlations (range)	0.70–0.84		0.70–0.86	
Descriptive statistics				
Mean *(SD)*	26.39 (12.07)		12.88 (6.22)	
Range of scores	16–80		8–40	
Skewness	1.34		1.41	
Kurtosis	1.43		1.47	

*** *p* < 0.001; * *p* < 0.05; † *p* = 0.05. Model fit indices for the exploratory factor analyses (EFA) were estimated on the first subsample (*n* = 377), while model fit indices for the confirmatory factor analyses (CFA) were estimated on the second subsample (*n* = 378). Reliability, descriptive statistics, and inter-item and item-total correlations were calculated on the total sample (*N* = 755). *CFI* Comparative Fit Index, *TLI* Tucker-Lewis index, *RMSEA* Root-Mean-Square Error of Approximation, *SRMR* Standardized Root-Mean-Square Residuals, *CI* confidence interval, *df* = degrees of freedom, *SD* = standard deviation

Model fit improved for the 8-item version of the PCFS, showing excellent fit for the unidimensional structure when compared to the 16-item version. Moreover, internal consistency in the 8-item PCFS remained high (though slightly decreased as item homogeneity was reduced). Therefore, the 8-item PCFS is recommended for use, and this brief version of the scale was further investigated. The final items are presented in Table 2 .

### Convergent and divergent validity of the Problematic Celebrity Fanship Scale (PCFS)

To examine the relationship between problematic celebrity fanship and related constructs, a CFA with covariates was performed. In this analysis, all relevant constructs were represented as latent variables and served as predictors of the latent variable for problematic celebrity fanship. The correlation matrix underlying the model is presented in Table 4, and the pattern of correlations supports the validity of the latent variable. Specifically, the results provide support for the convergent validity of problematic celebrity fanship, as it correlated moderately to strongly with psychological distress (*r* = 0.45; *p* < 0.001) and problematic Internet use (*r* = 0.51; *p* < 0.001), and weakly with problematic social media use (*r* = 0.14; *p* < 0.001), indicating that these constructs represent conceptually related maladaptive behaviors. It also showed a positive association with the two dimensions of celebrity worship (*r* = 0.53; *p* < 0.001 for the intense–pathological and *r* = 0. 47; *p* < 0.001 for the entertainment–social dimension), which reflects an excessive admiration towards a celebrity.

**Table 4:** Correlations between problematic celebrity fanship and related constructs

	1.	2.	3.	4.	5.	6.	7.	8.	9.
1. Problematic celebrity fanship	–								
2. Problematic Internet use	0.51***	–							
3. Problematic social media use	0.14***	0.26***	–						
4. Psychological distress	0.45***	0.48***	0.20***	–					
5. Entertainment–social celebrity worship	0.47***	0.22***	0.01	0.17***	–				
6. Intense–pathological celebrity worship	0.53***	0.41***	0.12**	0.39***	0.74***	–			
7. Parasocial relationship	0.18***	0.08*	−0.02	−0.06	0.62***	0.34***	–		
8. Self-concept clarity	−0.47***	−0.45***	−0.19***	−0.56***	−0.14**	−0.48***	0.12*	–	
9. Gender (*1* = men, *2* = women)	0.02	< 0.001	< 0.001	< 0.001	< 0.001	< 0.001	< 0.001	< 0.001	–
10. Age (years)	−0.05	< 0.001	< 0.001	< 0.001	< 0.001	< 0.001	< 0.001	< 0.001	0.04

*N* = 755. ***p < 0.001; **p < 0.01; *p < 0.05. Gender and age were observed variables, while all other variables were latent constructs in the CFA with covariates model. Missing data were handled using the Full Information Maximum Likelihood (FIML) method. The covariance coverage ranged from 90% to 100%

Furthermore, divergent validity was supported by the relatively weaker association with parasocial relationship (*r* = 0.18; *p* < 0.001), a construct representing more normative or non-pathological form of engagement with a celebrity. In addition, the negative correlation with self-concept clarity (*r* = −0.47; *p* < 0.001) and the negligible associations with gender and age (*p*s > 0.05) demonstrate that problematic celebrity fanship is empirically distinct from personality variables or demographic characteristics.

The CFA with covariates model provided additional evidence for the construct validity of problematic celebrity fanship by identifying the unique covariances of theoretically relevant predictors with the latent construct, while controlling for shared variance among them (see SM 6). Highly correlated explanatory variables were excluded to mitigate multicollinearity. The severity of problematic celebrity fanship was positively predicted by problematic Internet use (*β* = 0.27, *p* < 0.001), entertainment–social celebrity worship (*β* = 0.24, *p* < 0.001), and psychological distress (*β* = 0.15, *p* = 0.003), while negatively predicted by self-concept clarity (*β* = −0.23, *p* < 0.001). Parasocial relationship did not show a unique association with the construct (*β* = 0.05, *p* = 0.30), suggesting that parasocial bonds are not inherently maladaptive when other psychological factors are accounted for. Among demographic covariates, gender was a nonsignificant predictor (*p* > 0.05). Age showed a marginally significant association with an MLR estimator (*β* = −0.06, *p* = 0.06) and a very weak association with a WLSMV estimator (*β* = −0.17, *p* < 0.001), suggesting that problematic celebrity fanship may decrease with age. These results confirm that problematic celebrity fanship maintains its theoretically expected pattern of associations even when controlling for the interrelations among predictors, thus providing strong multivariate evidence of convergent and nomological validity. The covariates explained 41.1% of the variance in problematic celebrity fanship severity.

### Measurement invariance based on gender

Measurement invariance was tested with regard to gender (see Table 5), showing consistently excellent model fit indices for the PCFS in the baseline models and the configural, metric, and scalar models with increasingly constrained parameters. The chi-square difference test confirms the lack of significantly poorer fit in the case of increasingly restrictive models, and the changes observed in the more liberal fit indices can also be considered negligible, indicating that gender comparisons in PCFS scores are meaningful.

**Table 5:** Testing gender invariance on the Problematic Celebrity Fanship Scale (PCFS)

Model	χ^2^ (df)	CFI	TLI	RMSEA (90% CI)	SRMR	Model comparison	Δ χ^2^ (df)	Δ CFI	Δ TLI	Δ RMSEA	Δ SRMR
Baseline											
Men (*n* = 388)	28.698 (20)	0.993	0.990	0.033 (0.000–0.059)	0.019						
Women (*n* = 367)	37.482 (20)*	0.980	0.973	0.049 (0.023–0.073)	0.029						
Configural	66.294 (40)**	0.987	0.982	0.042 (0.023–0.059)	0.024						
Metric	73.759 (47)**	0.987	0.985	0.039 (0.020–0.055)	0.033	metric vs. configural	6.396 (7)	0.000	0.003	0.003	0.009
Scalar	81.683 (54)**	0.987	0.986	0.037 (0.019–0.052)	0.032	scalar vs. metric	5.593 (7)	0.000	0.001	0.002	0.001

** *p* < 0.01; * *p* < 0.05. *CFI* Comparative Fit Index, *TLI* Tucker-Lewis index, *RMSEA* Root-Mean-Square Error of Approximation, *SRMR* Standardized Root-Mean-Square Residuals, *CI* confidence interval, *df* = degrees of freedom

### Latent profile analysis to identify problematic celebrity fanship

LPA was performed on the PCFS, showing high entropy across different models, with decreasing AIC, BIC, and SSABIC values with the increasing number of profiles. The LMR test suggested that the three-profile model should be selected for further analysis based on the nonsignificant *p*-value for the four-profile model (see Table 6).

**Table 6:** Latent profile analysis (LPA) fit indices for the Problematic Celebrity Fanship Scale (PCFS)

Profiles (*n*)	AIC	CAIC	BIC	SSABIC	Entropy	LMR Test	*p*
1	16236.38	16326.41	16310.41	16259.61	–	–	–
2	12411.02	12542.69	12526.69	12447.31	0.97	3779.98	0.000
**3**	**11646.07**	**11825.38**	**11803.38**	**11695.41**	**0.98**	**770.04**	**0.003**
4	11277.71	11504.66	11476.66	11340.11	0.95	379.99	0.312
5	10536.53	10811.12	10777.12	10612.00	0.99	237.81	0.063
6	9369.40	9691.63	9651.63	9457.93	0.99	340.26	0.233

*AIC* Akaike information criterion, *CAIC* bias-corrected Akaike information criterion, *BIC* Bayesian information criterion, *SSABIC* sample-size adjusted Bayesian information criterion, *LMR Test* Lo–Mendell–Rubin Adjusted Likelihood Ratio Test; *p* = *p*-value for the LMR Test. The final model, selected based on the fit indices, is marked in **bold**

**Fig. 1:**
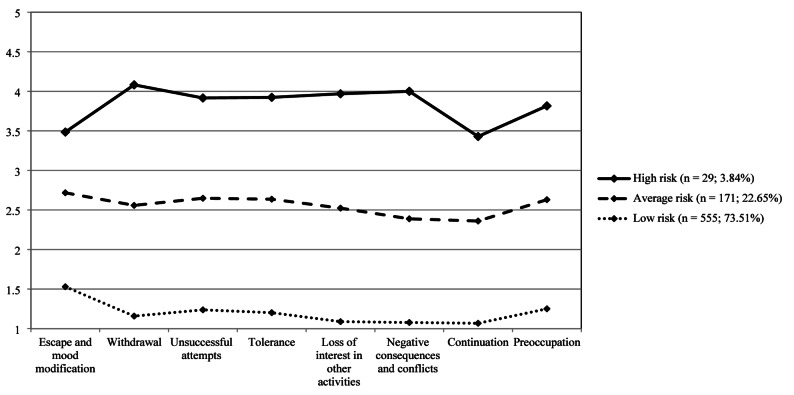
*P*roblematic Celebrity Fanship Scale (PCFS) item scores across the three latent profiles (high risk, average risk, low risk)

The three-profile model assigned participants to three categories: (1) individuals showing high-risk for problematic celebrity fanship (*n* = 29; 3.84%), with higher scores on all PCFS items, particularly on the items representing withdrawal, and negative consequences and conflicts; (2) individuals at average risk, scoring lower than the high-risk group on all items (*n* = 171; 22.65%), and (3) individuals at low risk (*n* = 555; 73.51%), having consistently low scores on all items (see Fig. 1). Differences between the three latent profile groups in key variables are presented in Table 7. In detail, the high-risk group showed higher levels of problematic celebrity fanship, celebrity worship, parasocial relationship, problematic Internet and social media use, psychological distress, and lower levels of self-concept clarity compared to the low-risk group. The average-risk group also yielded higher scores on these indicators than the low-risk group, except for self-concept clarity, on which the average-risk group had lower scores than the low-risk group, and parasocial relationship, in which there was no significant difference between the average-risk and the low-risk group. The high-risk group also yielded higher levels of problematic celebrity fanship and celebrity worship than the average-risk group.

**Table 7:** Group comparisons in study-variables across latent profiles based on the latent profile analysis (LPA)

Variable (range of the response scale) M (SD)	High-risk group (*n* = 29; 3.84%)	Average risk group (*n* = 171; 22.65%)	Low risk group (*n* = 555; 73.51%)	F	η^2^
Problematic celebrity fanship (8–40)	30.66 (3.83)a	20.47 (2.88)b	9.61 (2.12)c	2178.60***	0.85
Problematic Internet use (9–45)	19.07 (7.68)a	17.97 (7.04)a	11.95 (4.58)b	90.37***	0.20
Problematic social media use (6–30)	13.96 (4.21)a	14.45 (4.77)a	8.70 (3.48)b	143.69***	0.30
Psychological distress (0–3)	2.41 (2.11)a	2.28 (1.72)a	0.87 (1.29)b	73.50***	0.16
Celebrity worship (total; 7–35)	27.68 (5.72)a	23.33 (5.00)b	16.15 (6.06)c	137.10***	0.27
Entertainment–social celebrity worship (3–15)	12.55 (2.25)a	10.40 (2.50)b	8.68 (3.44)c	35.08***	0.09
Intense–pathological celebrity worship (4–20)	15.38 (3.94)a	12.93 (3.04)b	7.46 (3.55)c	213.93***	0.36
Parasocial relationship (1–5)	4.05 (0.82)a	3.64 (0.70)ab	3.47 (0.89)b	8.30***	0.02
Self-concept clarity (12–60)	36.92 (9.56)a	39.33 (7.96)a	47.84 (8.54)b	80.78***	0.18

*** *p* < 0.001. One-way ANOVA was conducted for group comparisons. To reduce the risk of Type I error, the *p*-value for multiple comparisons in this analysis was set at *p* < 0.01. Identical letters in the same row indicate nonsignificant (*p* > 0.01) difference between groups, while different letters indicate significant group differences (*p* < 0.01) according to the post-hoc Tukey test. *M* = mean, *SD* = standard deviation

The high-risk profile was used as a gold standard for subsequent estimations of sensitivity, specificity, PPV, NPV, and accuracy (see Table 8). A score of 26 is suggested as an optimal cutoff point for identifying individuals who show problematic levels of celebrity fanship. Based on this cutoff point, the vast majority of individuals with problematic celebrity fanship were identified correctly (96.6%), while only 0.4% were incorrectly classified as not having problematic celebrity fanship. Moreover, most individuals who did not show problematic levels of celebrity worship were also correctly identified (99.3%). Overall, on the unweighted fan sample (*N* = 755), 4.37% (*n* = 33) was classified as having problematic levels of celebrity fanship. These individuals scored higher on problematic Internet use and social media use measures, expressed higher levels of celebrity worship and parasocial relationship, and had weaker self-concept clarity compared to fans without problematic celebrity fanship. These differences were moderate-to-large with regard to psychological distress and parasocial relationship, and large with regard to all other variables (see Table 9). Prevalence rates estimated on the weighted sample were 1.44% in the Hungarian adult population aged 18–64 years (*N* = 2,000) and 2.34% in the subsample of Hungarian young adults aged 18–34 years (*N* = 1,001).

**Table 8:** Cutoff score thresholds for the problematic celebrity fanship scale (PCFS)

Cutoff score	True positive	True negative	False positive	False negative	Sensitivity (%)	Specificity (%)	PPV (%)	NPV (%)	Accuracy (%)
12	29	448	278	0	100	61.71	9.45	100	63.18
13	29	482	244	0	100	66.39	10.62	100	67.68
14	29	510	216	0	100	70.25	11.84	100	71.39
15	29	540	186	0	100	74.38	13.49	100	75.36
16	29	551	175	0	100	75.90	14.22	100	76.82
17	29	570	156	0	100	78.51	15.68	100	79.34
18	29	591	135	0	100	81.40	17.68	100	82.12
19	29	601	125	0	100	82.78	18.83	100	83.44
20	29	622	104	0	100	85.67	21.80	100	86.23
21	29	642	84	0	100	88.43	25.66	100	88.87
22	29	662	64	0	100	91.18	31.18	100	91.52
23	29	677	49	0	100	93.25	37.18	100	93.51
24	29	698	28	0	100	96.14	50.88	100	96.29
25	28	708	18	1	96.55	97.52	60.87	99.86	97.48
**26**	**28**	**721**	**5**	**1**	**96.55**	**99.31**	**84.85**	**99.86**	**99.21**
27	26	726	0	3	89.66	100	100	99.59	99.60
28	23	726	0	6	79.31	100	100	99.18	99.21
29	20	726	0	9	68.97	100	100	98.78	98.81

*PPV* positive predictive value, *NPV* negative predictive value. The final cutoff score is marked in **bold**. Scores on the PCFS can range from 8 to 40

**Table 9:** Group comparisons in study-variables across individuals with and without problematic celebrity fanship based on the cutoff score of 26 on the PCFS

Variable (range of the response scale) M (SD)	Individuals with problematic celebrity fanship (*n* = 33; 4.37%)	Individuals with average celebrity fanship (*n* = 722; 95.63%)	t	Cohen’s d
Problematic celebrity fanship (8–40)	30.15 (3.82)	12.09 (5.06)	−26.15***	4.03
Problematic Internet use (9–45)	19.23 (7.55)	13.28 (5.79)	−4.25***	0.88
Problematic social media use (6–30)	14.30 (4.33)	9.98 (4.49)	−5.16***	0.98
Psychological distress (0–3)	2.43 (2.16)	1.19 (1.51)	−3.26**	0.67
Celebrity worship (total; 7–35)	27.34 (5.42)	17.81 (6.57)	−8.08***	1.58
Entertainment–social celebrity worship (3–15)	12.36 (2.18)	9.07 (3.33)	−8.26***	1.17
Intense–pathological celebrity worship (4–20)	15.21 (3.81)	8.72 (4.14)	−8.84***	1.63
Parasocial relationship (1–5)	4.08 (0.76)	3.50 (0.85)	−3.81***	0.72
Self-concept clarity (12–60)	36.08 (9.64)	45.92 (9.08)	5.98***	1.05

***p < 0.001; ** *p* < 0.01. Independent-samples t-test was conducted for group comparisons. To reduce the risk of Type I error, the *p*-value for multiple comparisons was set at *p* < 0.01 in this analysis. *M* = mean, *SD* = standard deviation

## Discussion

There has been considerable research interest in excessive levels of celebrity admiration in the past few decades [1]. Existing measures either combine general and problematic levels of celebrity worship in a single measurement or lack strong theoretical background. The convenience samples used to estimate the occurrence of excessive celebrity worship also undermined the generalizability of past findings. To overcome these limitations, the present study developed a brief, psychometrically sound scale assessing problematic celebrity fanship within a behavioral addiction theoretical framework, using a sample of fans derived from a representative sample of adults. The 8-item unidimensional Problematic Celebrity Fanship Scale (PCFS) demonstrated good psychometric properties in terms of factor structure, reliability, and validity. Based on the suggested cutoff score of 26, 1.44% of individuals in the present, nationally representative sample of Hungarian adults showed problematic celebrity fanship. Problematic celebrity fanship was associated with problematic Internet use and social media use, psychological distress, and weaker self-concept clarity. The PCFS can assist further investigations to gain a more nuanced knowledge of the nature and dynamics of problematic engagement with celebrities.

The PCFS was constructed based on the DSM–5 criteria of gambling disorder, the suggested criteria for Internet gaming disorder (termed gaming disorder in the ICD-11; [30]), and the 6-component model of addictions by Griffiths [31], which have been generally used as a basis of scale development for self-report measures assessing behavioral addictions, such as problematic Internet use [25], social media use [23], and pornography use [55]. The 8-item unidimensional PCFS yielded excellent model fit indices, high reliability, and demonstrated gender invariance. Gender invariance in the present sample indicates that comparisons across men and women in problematic celebrity fanship can be meaningful using the PCFS, providing preliminary support for the generalizability of the scale to more diverse populations. Specifically, the scale measures similarly across gender-based groups according to the present findings, suggesting that the risk of measurement bias and inadequate comparisons based on gender are minimal.

After enabling meaningful gender-based comparisons, similar to the findings by He and Liu [22], no gender difference was found in PCFS scores. The PCFS was only weakly and inconsistently associated with age. Problematic celebrity fanship severity was moderately associated with problematic Internet use symptom severity and celebrity worship. Likewise, problematic celebrity fanship severity was moderately associated with psychological distress and weaker self-concept clarity, which is in line with decades of empirical evidence on the association of celebrity worship with symptoms of depression, anxiety, and generally poorer mental health [1, 17]. Moreover, results regarding self-concept clarity concur with previous findings indicating a negative relationship between celebrity worship and self-concept clarity [56] and integrity [14], in accordance with the Absorption–Addiction Model [2, 3]. However, problematic celebrity fanship severity was only weakly associated with problematic social media use severity and stronger parasocial relationships, suggesting that the construct of problematic celebrity fanship is more strongly associated with psychopathological constructs than one-sided, socio-emotional experiences on the healthy spectrum. Overall, these patterns are consistent with theoretical expectations suggesting that excessive celebrity involvement forms part of a broader constellation of dysregulated media use and emotional difficulties. Collectively, the present findings confirm that problematic celebrity fanship represents a unique maladaptive dimension of celebrity admiration rather than a mere extension of general fandom or demographic tendencies, supporting the validity of the PCFS.

Based on the suggested cutoff score of 26, the prevalence rate of problematic celebrity fanship was 1.4% in the Hungarian adult population (18–64 years of age), and 2.3% in the subsample of young adults (18–34 years of age). Prevalence rates below 5% are similar to those reported for other behavioral addictions using representative samples of European adults and adolescents (see [57–59]). For instance, Király et al. [59] reported prevalence rates ranging from 1.61% to 4.48% in relation to gaming disorder, while Bányai et al. [23] identified 4.5% of individuals at risk of problematic social media use. However, the present rates of problematic celebrity fanship are somewhat lower than the rates of celebrity worship found using the 7-item Celebrity Attitude Scale (4.5% among adults and 8.5% among young adults; [12]), which can be attributed to the broader spectrum of behaviors comprising the concept of celebrity worship (i.e., entertainment–social and intense–personal dimensions were merged when estimating prevalence rates for celebrity worship).

When comparing individuals with problematic levels of celebrity fanship to those with average-level celebrity fanship, moderate-to-large and large differences were found in the present study in terms of engagement with a favorite celebrity (i.e., celebrity worship and parasocial relationship), problem behaviors (i.e., problematic Internet and social media use), psychological distress, and self-concept clarity. Specifically, individuals exhibiting problematic levels of celebrity fanship reported much greater mental health difficulties and problem behaviors than fans with lower levels of engagement with a favorite celebrity. This result is in line with current empirical evidence showing that those affected by excessive celebrity worship [1, 12] and behavioral addictions [8] are generally more likely to experience mental health problems and other problem behaviors.

The present authors suggest using the term “celebrity fanship” for the construct under investigation instead of the generally used expression “celebrity worship” to draw a clear line between nonproblematic (i.e., healthy) and problematic engagement with celebrities. As “worship” indicates an excessive behavior, its use for the description of this comprehensive, multifaceted phenomenon (which could be problematic or nonproblematic) could be somewhat misleading [11]. The term “problematic celebrity fanship” refers to addictive levels of engagement with celebrities, which can occur in a small proportion of fans, while most fans appear to exhibit healthy enthusiasm and interest levels towards celebrities, as proposed by the Absorption–Addiction Model [2, 3]. Therefore, this conceptual clarification can possibly reduce the risk of overpathologizing everyday life activities [60], such as high but nonproblematic engagement with an admired celebrity, which may be part of the normal development process of identity formation during adolescence [61]. Although decades of research into fan engagement (e.g., [1, 2, 9, 10, 22]) has provided compelling evidence for the unique features of excessive celebrity admiration, enabling a more nuanced conceptualization and operationalization of the phenomenon, investigations in clinical settings are still lacking. Clinical samples would be needed for the broader exploration of functional impairment and the temporal stability of the problematic behavior, which are crucial for the confirmation of new behavioral addictions [60]. Indeed, addictive behaviors can be context-dependent and temporary [60]. In this regard, celebrities can become rapidly popular among specific groups (e.g., adolescents), but a short-term, intense immersion into the work, personal characteristics, and achievements of a celebrity should not necessarily be considered dysfunctional or problematic behavior. This enthusiasm is likely to decline fast, as was observed in relation to other rewarding, potentially addictive behaviors (e.g., video gaming; [60]). Although it is important to avoid the general pathologization of highly engaging behaviors, extending the theoretical framework and enabling a more specific and accurate measurement of a phenomenon that has been investigated for decades, and is known to be associated with psychological difficulties is needed to establish the foundations of more intense clinical investigations and potential harm reduction efforts.

Based on this theoretical background, drawing on the definition of entertainment–social celebrity admiration [2, 3], the authors of the present study define celebrity fanship as an emotional engagement and dedication towards a celebrity for their work, personal characteristics, performance, or achievements. Besides feelings of a personal connection with the celebrity (e.g., inspiration), such admiration can manifest in various behaviors, including following the celebrity’s updates and participating in fan activities (e.g., creating art, attending events, or joining fan communities). By contrast, based on the definition of behavioral addictions by Vieira et al. [18], problematic celebrity fanship can be defined as an excessive preoccupation with a celebrity, characterized by compulsive and impulsive behaviors, which can interfere with individuals’ everyday functioning, responsibilities, other leisure activities, and mental health.

The Absorption–Addiction Model [2, 3] describes the process of developing an excessive admiration towards a celebrity as a gradually increasing engagement and identification with a celebrity’s work, then personal life, feelings, and behavior. As a process model, the Absorption–Addiction Model suggests that potentially addictive levels of celebrity admiration include both behaviors typical to healthy admiration levels (e.g., individuals enjoy following the news about their favorite celebrity) and problematic levels (e.g., feeling an urge to learn the favorite celebrity’s personal habits). Therefore, while the Absorption–Addiction Model explains the underlying mechanism of developing an excessive admiration towards a celebrity, it may not provide a clear distinction between healthy and problematic levels of engagement, which could possibly enable the early recognition and prevention of excessive involvement. The present study drew a clearer line between healthy and problematic celebrity fanship by offering an assessment instrument that can identify individuals potentially at risk of experiencing mental health difficulties in relation to their excessive engagement with a celebrity. Indeed, the PCFS can assist early screening of problematic behavioral patterns and risk estimation, informing researchers and practitioners about problematic levels of celebrity fanship which may deserve further attention. Additionally, the present study could be a preliminary step towards developing the theoretical foundation of a potentially addictive behavior (i.e., problematic celebrity fanship), which has been integral part of the celebrity worship research tradition of the past few decades (see the Absorption–Addiction Model; [2, 3]). Indeed, unhealthy levels of celebrity admiration have been widely researched [62], and although studies found a considerable overlap between features of behavioral addictions (e.g., gambling addiction, problematic Internet and social media use; [19]) and celebrity worship, the potentially addictive levels of celebrity admiration have been investigated only recently within a behavioral addiction theoretical framework [22]. The present study contributed to this theoretical approach by providing a clear definition of problematic celebrity fanship, which is distinguishable from healthy celebrity fanship, and developed a valid and psychometrically sound assessment instrument to measure the construct accurately, based on the current literature on behavioral addictions.

### Limitations

Although the present study offered evidence on the validity of the PCFS, further research is needed to confirm its psychometric appropriateness and applicability in diverse populations (e.g., adolescents, other cultures). Further investigations are also needed to test gender invariance among individuals from different cultural backgrounds. Additionally, testing language-based invariance would be desirable to broaden the applicability of the PCFS to cross-cultural settings. Moreover, problematic celebrity fanship is not a diagnostic category; therefore, fans scoring above the suggested cutoff point on the PCFS should be considered at possible risk of problematic engagement with a celebrity, which needs further clinical exploration. The PCFS is not appropriate for clinical diagnostic purposes, and its usability in clinical settings still warrants empirical evidence. Considering the nature of the construct and the sample characteristics, the prevalence of problematic celebrity fanship estimated in the present study was compared to the prevalence rates of behavioral addictions and excessive celebrity worship that were estimated using nationally representative samples. However, future research on clinical samples should extend knowledge on the comorbidity of other disorders (e.g., pathological levels of depression, narcissism, and obsessive-compulsive disorder) with problematic celebrity fanship. Previous studies have found only weak associations of the problematic dimensions of celebrity worship with personality traits, obsessive-compulsive disorder [13, 14], and lower self-esteem [63]. Future research on the association of problematic celebrity fanship with more specific manifestations of disorders (e.g., relationship obsessive-compulsive disorder; 16) may provide new insights into the phenomenon. In relation to this concern, it should be noted that the present study used a cross-sectional design. Therefore, causal inferences or assumptions regarding directionality cannot be drawn from the present data. Finally, although prevalence rates were calculated on a weighted dataset, which allows for drawing broader conclusions regarding the Hungarian adult population, the purpose of this study was to develop a scale that can be administered only for individuals having a favorite celebrity. Therefore, the scale development process was conducted on an unweighted dataset of the subsample of fans, which limits the generalizability of the scale properties and correlates to the whole population of fans. Although a nonprobability (quota) sampling method was applied, and the total sample was representative of the Hungarian adult population with regard to gender, age, location, and size of residence, there may be further sociodemographic characteristics for which the present sample may not be representative (e.g., occupation, socioeconomic status), reducing the generalizability of the findings. Future research should also explore possibilities for further generalization of the concept and measurement of fanship, considering musical bands or sports teams as objects of admiration instead of individuals.

### Implications and future directions

The present study offered a brief, concise, but comprehensive assessment instrument with sound psychometric properties to measure problematic celebrity fanship. Therefore, this study contributes to the literature of behavioral addictions by introducing the concept of problematic celebrity fanship, and broadens the range of valid and reliable assessment tools measuring potentially addictive behaviors. Addressing the limitations of previous studies, this study also differentiates between definitions of nonproblematic (healthy) and problematic celebrity fanship, and focuses specifically on the conceptualization and operationalization of the problematic dimension of engagement with a celebrity. The present findings supported the psychometric appropriateness of the PCFS to assess problematic celebrity fanship severity in a diverse adult population, enabling meaningful gender-based comparisons. This is particularly important since previous studies often sampled undergraduate students [11]. Future studies should extend this finding to an international context, considering that men and women may interpret and experience problematic celebrity fanship differently in other cultures. To enhance the scale’s cross-cultural applicability, the translation and validation of the PCFS in diverse cultural settings would also be needed. This effort could facilitate the estimation and comparison of prevalence rates across countries. The extension of investigation to other populations potentially more susceptible to problematic celebrity fanship (e.g., adolescents) and conducting research in clinical settings could provide valuable insights into associated harms and vulnerable groups. Finally, exploring the divergent and convergent associations of mental health indicators, personality traits, quality of social relationships, and other behavioral addictions with problematic celebrity fanship could provide a more nuanced understanding of the distinctive features of problematic celebrity fanship, contributing to a clearer conceptual differentiation between this construct and other addictive behaviors.

## Conclusions

In summary, the present study defined and operationalized problematic celebrity fanship in the current theoretical framework of behavioral addictions. Problematic celebrity fanship can be described as an excessive preoccupation with a celebrity, which manifests in behaviors that can impair everyday life functioning in various areas, including professional and social life, and other free time activities. According to the present findings, 1.44% of Hungarian adults in the present, nationally representative sample showed problematic levels of celebrity fanship. These individuals exhibited substantially higher levels of psychological distress, problematic Internet and social media use severity, and lower self-concept clarity compared to individuals with average levels of celebrity fanship. The Problematic Celebrity Fanship Scale (PCFS) can assist research and practice by providing a screening tool to identify fans at higher risk of experiencing mental health difficulties associated with excessive engagement with an admired celebrity. Overall, this study could be a step towards the conceptualization and recognition of problematic celebrity fanship as an addictive behavior.

## Supplementary Information

Supplementary Material 1

## Data Availability

The dataset used in the current study is available from the corresponding author on reasonable request.

## Abbreviations

AIC: Akaike information criterion
BA: Bachelor degree
BIC: Bayesian information criterion
BSMAS: Bergen Social Media Addiction Scale
CAIC: Bias-corrected Akaike information criterion
CAPI: Computer-assisted personal interviewing
CASI: Computer-assisted self-interviewing
CAS: Celebrity Attitude Scale
CFA: Confirmatory factor analysis
CFI: Comparative fit index
CI: Confidence interval
DASS: Depression, Anxiety, and Stress Scale
DSM: Diagnostic and Statistical Manual of Mental Disorders
DF: Degrees of freedom
EFA: Exploratory factor analysis
FIML: Full information maximum likelihood
ICD: International Classification of Diseases
KMO: Kaiser-Meier-Olkin
LPA: latent profile analysis
LMR: Lo-Mendell-Rubin adjusted likelihood ratio
M: Mean
MA: Master’s degree
MLR: Robust maximum likelihood estimator
N: Number
NPV: Negative predictive value
PCFS: Problematic Celebrity Fanship Scale
PIUQ: Problematic Internet Use Questionnaire
POGQ: Problematic Online Gaming Questionnaire
PPV: Positive predictive value
PSI: Parasocial Interaction Scale
RMSEA: Root-mean-square error of approximation
SD: Standard deviation
SM: Supplementary materials
SRMR: Standardized root-mean-square residuals
SSABIC: Sample-size adjusted Bayesian information criterion
SSC: Self-Concept Clarity Scale
TLI: Tucker-Lewis index
WLSMV: Weighted least squares mean and variance adjusted estimator
