# Supplementary material for: When fandom becomes a problem: Development of the Problematic Celebrity Fanship Scale using a representative adult sample
Source: BMC Psychol. 2026 Feb 4;14:312. doi: 10.1186/s40359-026-04064-w (PMC12964916; doi:10.1186/s40359-026-04064-w)
Supplement: Supplementary file 1 — Supplementary Material 1 [file 40359_2026_4064_MOESM2_ESM.docx]

**Supplementary materials (SM)**

**SM 1**

***Problematic Celebrity Fanship Scale (PCFS)***

Please think back to the past 12 months and indicate on the following scale how often you experienced these things in relation to your favorite celebrity.

|  | Never | Rarely | Sometimes | Often | Almost always/  always |
| --- | --- | --- | --- | --- | --- |
| 1. My interest in my favorite celebrity has helped me forget about my real-life problems. | 1 | 2 | 3 | 4 | 5 |
| 1. I got upset when something prevented me from seeing/hearing my favorite celebrity. | 1 | 2 | 3 | 4 | 5 |
| 1. I have been unsuccessful at spending less time following my favorite celebrity’s life. | 1 | 2 | 3 | 4 | 5 |
| 1. I have increased my time spent following the details of my favorite celebrity’s life. | 1 | 2 | 3 | 4 | 5 |
| 1. I had trouble getting interested in anything else besides my favorite celebrity. | 1 | 2 | 3 | 4 | 5 |
| 1. My interest in my favorite celebrity has led to conflicts with others (e.g., parents, classmates). | 1 | 2 | 3 | 4 | 5 |
| 1. Despite being aware that my celebrity interest was negatively influencing my work/studies, I couldn’t stop it. | 1 | 2 | 3 | 4 | 5 |
| 1. When I thought about my favorite celebrity, I forgot about everything else. | 1 | 2 | 3 | 4 | 5 |

**SM 2**

*Factor loadings of the initial, 16-item version of the Problematic Celebrity Fanship Scale (PCFS) based on exploratory (EFA) and confirmatory factor analysis (CFA) on randomly half-split samples of the total sample (N = 755)*

| Item content | Component | EFA  (*n* = 377) | EFA  (WLSMV estimator)  (*n* = 377) | CFA  (MLR estimator)  (*n* = 378) | CFA  (WLSMV estimator)  (*n* = 378) |
| --- | --- | --- | --- | --- | --- |
| 1. My interest in my favorite celebrity has negatively affected my academic performance or my work. | negative consequences and conflicts | 0.678 | 0.802 | 0.701 | 0.835 |
| 2. I unsuccessfully tried to reduce my preoccupation with the life of my favorite celebrity. | unsuccessful attempts | 0.772 | 0.854 | 0.756 | 0.849 |
| **3. My interest in my favorite celebrity has helped me forget about my real-life problems.** | **escape and mood modification** | 0.675 | 0.781 | 0.568 | 0.689 |
| **4. I got upset when something prevented me from seeing/hearing my favorite celebrity.** | **withdrawal** | 0.829 | 0.893 | 0.807 | 0.885 |
| 5. I fantasized about how great it would be to meet my favorite celebrity. | preoccupation | 0.687 | 0.788 | 0.594 | 0.725 |
| **6. I have been unsuccessful at spending less time following my favorite celebrity’s life.** | **unsuccessful attempts** | 0.834 | 0.893 | 0.798 | 0.870 |
| 7. Seeing/hearing my favorite celebrity helped me relieve everyday stress. | escape and mood modification | 0.648 | 0.751 | 0.630 | 0.741 |
| 8. I became restless or irritable when I couldn’t watch/hear my favorite celebrity for as long as I wanted. | withdrawal | 0.833 | 0.894 | 0.829 | 0.906 |
| **9. I have increased my time spent following the details of my favorite celebrity’s life.** | **tolerance** | 0.814 | 0.885 | 0.837 | 0.906 |
| **10. I had trouble getting interested in anything else besides my favorite celebrity.** | **loss of interest in other activities** | 0.824 | 0.891 | 0.865 | 0.924 |
| **11. My interest in my favorite celebrity has led to conflicts with others (e.g., parents, classmates).** | **negative consequences and conflicts** | 0.814 | 0.888 | 0.841 | 0.914 |
| **12. Despite being aware that my celebrity interest was negatively influencing my work/studies, I couldn’t stop it.** | **continuation** | 0.813 | 0.892 | 0.836 | 0.914 |
| 13. I knew that my celebrity interest caused problems in my personal life, but I couldn’t change it. | continuation | 0.792 | 0.880 | 0.845 | 0.919 |
| 14. Sometimes interest in my favorite celebrity caused me to sleep less or forget to eat. | loss of interest in other activities | 0.791 | 0.877 | 0.866 | 0.931 |
| 15. I fantasized more and more about my favorite celebrity. | tolerance | 0.845 | 0.927 | 0.842 | 0.887 |
| **16. When I thought about my favorite celebrity, I forgot about everything else.** | **preoccupation** | 0.844 | 0.916 | 0.814 | 0.870 |

*Note.* Items retained for the short, 8-item version of the final scale are marked in **bold.** MLR = robust maximum likelihood estimator; WLSMV = weighted least squares mean and variance adjusted estimator. Model fit indices for the EFA with a WLSMV estimator were χ^2^ = 354.709, *df* = 104, *p* < 0.001; CFI = 0.985, TLI = 0.982, RMSEA = 0.080 (90% CI = 0.0.71; 0.089), eigenvalue was >1 (12.049, respectively) only for the unidimensional structure. Model fit for the CFA with a WLSMV estimator was initially unsatisfactory (*χ^2^* = 430.797, *df* = 104, *p* < 0.001; CFI = 0.982, TLI = 0.979, RMSEA = 0.091 [90% CI = 0.082; 0.100]). After inspection of the modification indices, two error covariances were added to the model (between item 3 and item 7; and between item 15 and item 16), which resulted in an improved model fit (*χ^2^* = 40.160, *df* = 20, *p* < 0.001; CFI = 0.986, TLI = 0.984, RMSEA = 0.080 [90% CI = 0.071; 0.089]).

**SM 3**

*Factor loadings and model fit indices of the exploratory (EFA) and confirmatory (CFA) factor analysis of the 8-item version of Problematic Celebrity Fanship Scale (PCFS) using a weighted least squares mean and variance adjusted (WLSMV) estimator*

| **Item** | **Factor loadings** | |
| --- | --- | --- |
|  | EFA  (*n* = 377) | CFA  (*n* = 378) |
| 1. My interest in my favorite celebrity has helped me forget about my real-life problems. | 0.436 | 0.663 |
| 2. I got upset when something prevented me from seeing/hearing my favorite celebrity. | 0.623 | 0.887 |
| 3. I have been unsuccessful at spending less time following my favorite celebrity’s life. | 0.591 | 0.868 |
| 4. I have increased my time spent following the details of my favorite celebrity’s life. | 0.615 | 0.902 |
| 5. I had trouble getting interested in anything else besides my favorite celebrity. | 0.615 | 0.933 |
| 6. My interest in my favorite celebrity has led to conflicts with others (e.g., parents, classmates). | 0.666 | 0.909 |
| 7. Despite being aware that my celebrity interest was negatively influencing my work/studies, I couldn’t stop it. | 0.556 | 0.919 |
| 8. When I thought about my favorite celebrity, I forgot about everything else. | 0.576 | 0.874 |
| **Model fit indices** |  |  |
| *χ^2^* (*df*) | 43.194 (20) | 40.160 (20) |
| *p* | 0.002 | 0.005 |
| CFI | 0.997 | 0.997 |
| TLI | 0.996 | 0.996 |
| RMSEA (90% CI) | 0.055  (0.033; 0.078) | 0.052  (0.028; 0.075) |

*Note.* The eigenvalue was > 1 only for the unidimensional structure (6.386, respectively) in the EFA. CFI = Comparative Fit Index, TLI = Tucker-Lewis index, RMSEA = Root-Mean-Square Error of Approximation, CI = confidence interval, *df* = degrees of freedom.

**SM 4**

*Unstandardized factor loadings and residual variances for the confirmatory factor analysis (CFA) of the Problematic Celebrity Fanship Scale (PCFS)*

| **Item number** | **MLR estimator** | | | | **WLSMV estimator** |  |
| --- | --- | --- | --- | --- | --- | --- |
|  | **Unstandardized factor loadings** | **Standardized factor loadings** | **Unstandardized residual variances** | **Standardized residual variances** | **Unstandardized**  **factor loadings** | |
| 1. | 0.580 | 0.541 | 0.815 | 0.708 | 0.663 | |
| 2. | 0.745 | 0.810 | 0.291 | 0.344 | 0.887 | |
| 3. | 0.797 | 0.796 | 0.366 | 0.366 | 0.868 | |
| 4. | 0.811 | 0.843 | 0.268 | 0.289 | 0.902 | |
| 5. | 0.790 | 0.877 | 0.188 | 0.231 | 0.933 | |
| 6. | 0.727 | 0.833 | 0.233 | 0.306 | 0.909 | |
| 7. | 0.658 | 0.843 | 0.176 | 0.290 | 0.919 | |
| 8. | 0.765 | 0.801 | 0.327 | 0.359 | 0.874 | |

*Notes. N* = 378. Item number indicates the order of items presented in SM 2. MLR = robust maximum likelihood estimator; WLSMV = weighted least squares mean and variance adjusted estimator.

**SM 5**

*Item-level descriptive statistics of the Problematic Celebrity Fanship Scale (PCFS)*

| **Item** | **Mean** | **SD** | **Skewness** | **Kurtosis** | **Item response distribution (*n*, %)** |
| --- | --- | --- | --- | --- | --- |
| 1. My interest in my favorite celebrity has helped me forget about my real-life problems. | 1.88 | 1.08 | 0.86 | -0.50 | 1 = never (401; 53.25%)  2 = rarely (127; 16.87%)  3 = sometimes (150; 19.92%)  4 = often (68; 9.03%)  5 = almost always/always (7; 0.9%) |
| 2. I got upset when something prevented me from seeing/hearing my favorite celebrity. | 1.59 | 0.96 | 1.55 | 1.49 | 1 = never (504; 66.93%)  2 = rarely (109; 14.48%)  3 = sometimes (95; 12.62%)  4 = often (36; 4.78%)  5 = almost always/always (9; 1.20%) |
| 3. I have been unsuccessful at spending less time following my favorite celebrity’s life. | 1.66 | 0.99 | 1.37 | 0.95 | 1 = never (470; 62.50%)  2 = rarely (125; 14.49%)  3 = sometimes (109; 14.49%)  4 = often (38; 5.05%)  5 = almost always/always (10; 1.33%) |
| 4. I have increased my time spent following the details of my favorite celebrity’s life. | 1.63 | 0.96 | 1.35 | 0.80 | 1 = never (478; 63.31%)  2 = rarely (128; 16.95%)  3 = sometimes (103; 13.64%)  4 = often (41; 5.43%)  5 = almost always/always (5; 0.66%) |
| 5. I had trouble getting interested in anything else besides my favorite celebrity. | 1.53 | 0.91 | 1.68 | 1.96 | 1 = never (519; 69.48%)  2 = rarely (107; 14.32%)  3 = sometimes (83; 11.11%)  4 = often (32; 4.28%)  5 = almost always/always (6; 0.80%) |
| 6. My interest in my favorite celebrity has led to conflicts with others (e.g., parents, classmates). | 1.49 | 0.89 | 1.84 | 2.60 | 1 = never (538; 71.93%)  2 = rarely (98; 13.10%)  3 = sometimes (76; 10.16%)  4 = often (29; 3.88%)  5 = almost always/always (7; 0.94%) |
| 7. Despite being aware that my celebrity interest was negatively influencing my work/studies, I couldn’t stop it. | 1.45 | 0.82 | 1.75 | 2.20 | 1 = never (535; 72.01%)  2 = rarely (105; 14.13%)  3 = sometimes (78; 10.50%)  4 = often (23; 3.10%)  5 = almost always/always (2; 0.27%) |
| 8. When I thought about my favorite celebrity, I forgot about everything else. | 1.67 | 0.94 | 1.28 | 0.81 | 1 = never (423; 59.00%)  2 = rarely (153; 21.34%)  3 = sometimes (103; 14.37%)  4 = often (32; 4.46%)  5 = almost always/always (6; 0.84%) |

*Notes.* *SD* = standard deviation. Item scores can range from 1 to 5. The count of missing values were 2 for item 1 and item 2; 3 for item 3; 8 for item 5; 7 for item 6; 12 for item 7; and 38 for item 8.

**SM 6**

*Confirmatory factor analysis (CFA) with covariates indicating the predictive power of correlates of problematic celebrity fanship severity (N = 755)*

|  | **Outcome: Problematic celebrity fanship severity** | | | | | |
| --- | --- | --- | --- | --- | --- | --- |
|  | **MLR estimator** | | | **WLSMV estimator** | | |
| **Predictors** | ***β*** | ***SE*** | ***p*** | ***β*** | ***SE*** | ***p*** |
| Problematic Internet use | 0.27 | 0.05 | <0.001 | 0.28 | 0.04 | <0.001 |
| Psychological distress | 0.15 | 0.05 | 0.003 | 0.17 | 0.05 | <0.001 |
| Entertainment–social celebrity worship | 0.24 | 0.05 | <0.001 | 0.29 | 0.06 | <0.001 |
| Parasocial relationship | 0.05 | 0.04 | 0.30 | 0.04 | 0.05 | 0.42 |
| Self-concept clarity | -0.23 | 0.04 | <0.001 | -0.24 | 0.04 | <0.001 |
| Gender | 0.005 | 0.03 | 0.86 | -0.02 | 0.04 | 0.59 |
| Age | -0.06 | 0.03 | 0.06 | -0.17 | 0.04 | <0.001 |

*Note.* Gender and age were observed variables, while all other variables in the model were defined as latent variables. Due to the high correlations between constructs of entertainment–social celebrity worship and intense–pathological celebrity worship (see Table 4) and the significant overlap between problematic Internet and social media use, the variables of problematic social media use and intense–pathological celebrity worship were excluded from the present analysis for interpretability. MLR = robust maximum likelihood estimator; WLSMV = weighted least squares mean and variance adjusted estimator, SE = standard error. R^2^ was 41.1% for the model with an MLR estimator and 54.0% for the model with a WLSMV estimator. Model fit using an MLR estimator (*χ^2^* = 2666.264, *df* = 1,208, *p* < 0.001; CFI = 0.925, TLI = 0.921, RMSEA = 0.040 [90% CI = 0.038; 0.042]), SRMR = 0.055) and a WLSMV estimator (*χ^2^* = 3327.871, *df* = 1,208, *p* < 0.001; CFI = 0.963, TLI = 0.961, RMSEA = 0.048 [90% CI = 0.046; 0.050]).

**SM 7**

*Testing gender invariance on the Problematic Celebrity Fanship Scale (PCFS) using a weighted least squares mean and variance adjusted* (*WLSMV) estimator*

| **Model** | χ^2^ (*df*) | CFI | TLI | RMSEA  (90% CI) | Model comparison | Δ χ^2^ (*df*) | Δ CFI | Δ TLI | Δ RMSEA |
| --- | --- | --- | --- | --- | --- | --- | --- | --- | --- |
| Baseline |  |  |  |  |  |  |  |  |  |
| Men  (*n* = 388) | 31.068 (20) | 0.999 | 0.998 | 0.038  (0.000–0.062) |  |  |  |  |  |
| Women  (*n* = 367) | 52.034 (20)*** | 0.995 | 0.993 | 0.066  (0.044–0.088) |  |  |  |  |  |
| Configural | 83.227 (40)*** | 0.997 | 0.996 | 0.054  (0.037–0.070) |  |  |  |  |  |
| Scalar | 91.435 (70)* | 0.999 | 0.999 | 0.028  (0.005–0.044) | scalar vs. configural | 25.635 (30) | 0.002 | 0.003 | 0.026 |

*Notes*. ** *p* < 0.01; * *p* < 0.05. Although the Δ RMSEA is higher than the suggested level of ≤ 0.015, the Δ χ^2^ is nonsignificant, indicating no significant change in the model fit across the scalar model and the configural model. CFI = Comparative Fit Index, TLI = Tucker-Lewis index, RMSEA = Root-Mean-Square Error of Approximation, CI = confidence interval, *df* = degrees of freedom.
